# Nutritional Behaviors, Vitamin Supplementation and Physical Activity among Polish Adults during the COVID-19 Pandemic

**DOI:** 10.3390/nu14020331

**Published:** 2022-01-13

**Authors:** Bogna Gryszczyńska, Magdalena Budzyń, Joanna Grupińska, Magdalena Paulina Kasprzak, Agnieszka Gryszczyńska

**Affiliations:** 1 Chair and Department of Medical Chemistry and Laboratory Medicine, Poznan University of Medical Sciences, 60-806 Poznan, Poland; magdalenabudzyn@ump.edu.pl (M.B.); jgrupinska@ump.edu.pl (J.G.); magdarut@ump.edu.pl (M.P.K.); 2 Greater Poland Cancer Centre, Hospital Pharmacy, 61-866 Poznan, Poland; 3 Institute of Natural Fibres and Medicinal Plants, National Research Institute, 60-630 Poznan, Poland; agnieszka.gryszczynska@iwnirz.pl

**Keywords:** COVID-19 pandemic, eating habits, vitamin supplementation, pickled food consumption, physical activity, daily lifestyle

## Abstract

The COVID-19 pandemic and its consequences, including social isolation, movement restrictions and work instability have altered many people’s nutritional behaviors and daily lifestyle. The aim of the study was to evaluate the influence of the COVID-19 pandemic on selected eating habits, physical activity and daily lifestyle changes of Polish adults (*n* = 145). The self-designed and anonymous questionnaire was available online from the 1 May 2021 to the 15 May 2021. In general, 60% of respondents declared that the COVID-19 pandemic did not affect their dietary habits, whereas 26% of surveyed individuals answered in the affirmative. The effect of the COVID-19 pandemic on changing dietary habits was differentiated by age (Pearson’s χ^2^ = 12.604; *p* = 0.0134). The number of meals consumed by respondents per day differed across gender groups (Pearson’s χ^2^ = 9.653; df = 4; *p* = 0.0466). An increase in body weight during the COVID-19 pandemic was reported by 43% of women and 7.6% of surveyed men. Additionally, hybrid working women declared most often an increase in body mass independent of age, education level and living place. Moreover, the majority of respondents who reported the effect of the pandemic on changing dietary habits also declared more frequent sweets consumption. The study revealed that respondents who stated more frequent sweets consumption during the COVID-19 pandemic were more likely associated with an increase in body mass (OR = 6.75, 95% CI, 6.75–91.25). No increase in the consumption of vitamin D, C and Mg supplements and pickled products was found.

## 1. Introduction

At the beginning of 2020, when some vague news about the coronavirus disease induced by SARS-CoV-2 reached Poland, it did not cause much commotion in society [1,2,3]. The rapid spread of the virus outside the Asian continent and finally the first case of the disease confirmed in Poland on the 4th of March 2020 changed many Poles’ lives diametrically [4,5]. The number of clients in the grocery stores was limited. That time was hard for everyone without exception, not only because of lockdown, but also because of work and financial instability, COVID-19 cases among our families and friends, movement restrictions and many other factors. 

The multitude of information, often mutually exclusive, has led to disorientation, fear and panic during the first wave (Spring 2020) and the second wave (Fall 2020) of the pandemic in Poland. Moreover, the extremely stressful situation of quarantine during each wave of the COVID-19 pandemic affects eating patterns [6,7]. Since the outbreak of the pandemic, the access to fresh food has been restricted, due to grocery shopping hours restrictions and clients limitations. People who had to change their workstyle spent more time at home and quite often limited their physical activity as well [8,9,10]. The isolation and easy access to so-called comfort foods, rich in sugar, led to their use to help decrease stress by increasing serotonin levels [8,11]. On the other hand, it could have been the best time for some positive habits, including developing cooking skills, restricting calories intake and starting physical activity at home [10].

The third COVID-19 wave in Poland took place in March and April 2021.The number of the new infected has been fluctuating daily, at around 20,000–35,000 cases [12,13]. The pessimistic frame of mind was affected not only because of the daily number of deaths or extended restrictions, but also because of the delay of vaccine delivery. The fourth wave of the COVID-19 pandemic has started between November and December in Poland. According to medical specialists, the fifth wave of the coronavirus will hit its peak in mid-January of 2022.

In the past two years, numerous survey studies were focused on the effect of the pandemic on social contact, movement restriction on nutrition behaviors, mental and health conditions, shopping frequency, education, alcohol drinking and cigarette smoking and many other factors in different study groups, starting with the youngest and ending with seniors [6,10,14]. The purpose of our study was to test the hypothesis that the COVID-19 pandemic had an effect on nutritional habits, vitamin supplementation, physical activity and lifestyle of surveyed respondents across age, gender, occupation, education level and living place groups. 

The aim of the present study was to evaluate the effect of the COVID-19 pandemic on the daily changes of food consumption, the body weight changes, physical activity and lifestyle was evaluated by using the self-designed cross-sectional online questionnaire (https://forms.office.com/r/bwGMA83kht, accessed on 3 November 2021). The questionnaire also consisted of the questions related to the effect of the COVID-19 pandemic on food consumption such as sweets, vegetables and sweet drinks and the supplementation of vitamin D, C and Mg. Knowing the side effects of the pandemic on societies may allow specifying more appropriate strategies to minimalize or control the spread of the epidemic [15,16]. Moreover, the results of the present study may help the dietitians and psychologists to understand their significant role in helping society to cope with the far-reaching effects of the COVID-19 pandemic. Both the results and our individual experiences may be helpful in case of any new pandemics in the future.

## 2. Material and Methods

The self-designed questionnaire was constructed to evaluate the effect of the COVID-19 pandemic on nutritional habits, physical activity and lifestyle. Additionally, some questions were related to mental and emotional condition, willingness to get a COVID-19 vaccination, shopping frequency, pro-ecological activity and consumers’ habits in Poland. However, such aspects are not discussed in the present manuscript. 

To evaluate the effect of the COVID-19 pandemic on consumers’ nutritional habits, physical activity and daily lifestyle in Poland, the survey study was designed as follows:

- The self-designed questionnaire, composed of closed-ended questions was constructed;
- The survey study was anonymous and voluntary;
- The potential respondents were contacted one time;
- The questions were multiple-choice, with more than one answer possible in some cases, such as in the mental and emotional conditions section;
- The questionnaire was composed of 37 questions divided into four sections:
  Section 1: Demographic questions consisting of seven queries;
  Section 2: Dietary habits and lifestyle disputes composed of 14 questions;
  Section 3: Six questions relating to the mental and emotional conditions. This section includes the question about the intention to receive a COVID-19 vaccine (not discussed in the present manuscript);
  Section 4: Inquiries relating to grocery shopping behaviors and pro-ecologic behaviors during the COVID-19 pandemic composed of 10 questions (not discussed in the present manuscript);
- The link to survey was administered via social media and was sent to the University of Medical Sciences workers by e-mails with the request for publication. The online research allowed to ensure safety of respondents under the pandemic conditions;
- The link to questionnaire was active from the 1 May 2021 to the 15 May 2021;
- The questionnaire was addressed to Polish females and males aged >18 years old. There were no exclusion criteria implemented, except the underage.

Within two weeks, 150 respondents answered the questions. The preliminary analysis showed that five questionnaires were filled partially and those were excluded from further analysis.

The purpose of our study was to test the hypothesis that the COVID-19 pandemic had an effect on nutritional habits, physical activity and daily lifestyle of surveyed respondents across age, gender, occupation, education level and living place groups.

The study hypothesis was verified based on the questions related to:

1. Whether the COVID-19 pandemic in Poland resulted in changes of workstyles and, if so, whether it affected Poles’ nutritional habits;
2. Whether lockdown during first, second and third waves of coronavirus in Poland resulted in daily changes of food consumption and physical activity resulting in weight changes;
3. Whether the COVID-19 pandemic resulted in lowering, increasing or did not affect sweets, fast foods, sweet drinks, energy drinks, fruits, vegetables and fish consumption at all;
4. Whether the news appearing in media, relating to the high hopes for the possible vitamin D protective effect against COVID-19, resulted in an increased consumption of that vitamin. We also asked respondents about the vitamin C and Mg supplementation, as well as pickled food consumption.

The statistical analysis was conducted using Statistica v 13.1 (StatSoft Inc., Tulsa, OK, USA) and GraphPad Prism version 9 (GraphPad Software, San Diego, CA, USA). The Pearson chi-square statistic test was used to evaluate tests of independence. The Pearson chi-square values (denoted as Pearson’s χ^2^), degrees of freedom (df) and *p* values were collected in the manuscript. Additionally, in order to provide greater statistical power and to estimate the independent influence of selected factors on analyzed parameters, multiple linear regression was carried out in the studied group. Moreover, multiple regression models were built to analyze the association between potential determinants and positive or negative changes. The odds ratio (OR) and 95% confidence interval (CI) were calculated. In all cases, *p*-value ≤ 0.05 was considered statistically significant.

## 3. Results

### 3.1. Survey Respondents’ Characteristic

**Figure 1:**
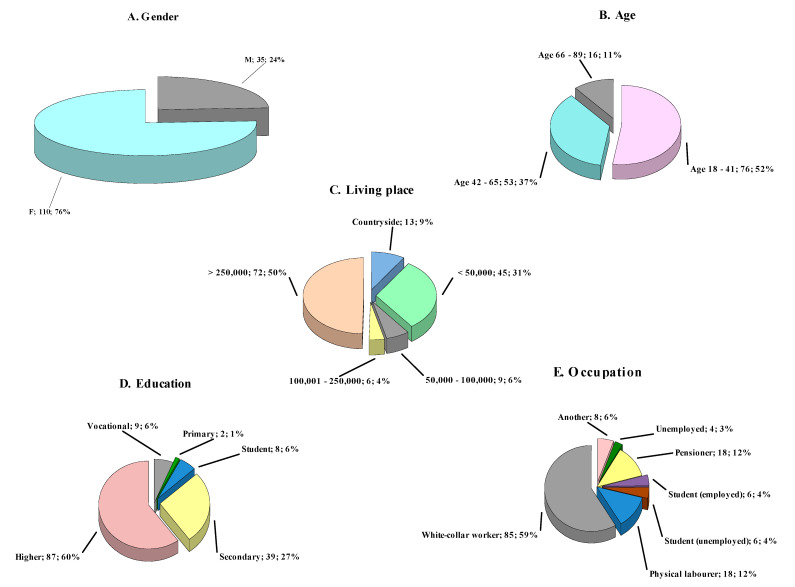
(**A**) Gender of questioned individuals. The segment description includes the gender (F = female; M = male), followed by the respondents’ number in this gender and percentage of total respondents, respectively. (**B**) Age of surveyed individuals. The segment description includes the respondents’ number aged in this range, followed by the percentage of the total respondents. (**C**) Living place of questioned participants. The segment description includes the city population, followed by the number of respondents inhabiting a given city and percentage of total respondents, respectively. (**D**) Education level of interviewees. The segment description includes the education level, followed by the number of respondents with that education stage and the percentage of total respondents, respectively. (**E**) Occupation of surveyed individuals. The segment description includes the employment, followed by the number of respondents who work in this profession and the percentage of total respondents, respectively.

One hundred and forty-five respondents answered the questionnaire thoroughly. In the present study, 110 females and 35 males answered the questions (Figure 1A). Most respondents were aged 18–41 (79 individuals), followed by aged 42–65 (53 individuals) and aged 66–89 years (16 individuals) respectively (Figure 1B). The analysis of questionnaires shows that most respondents live in cities with more than 250,000 inhabitants, while the least with 100,000 to 250,000 residents (Figure 1C). The figure shows that 9% of the studied population reside in the countryside. The education level breakdown of respondents is presented in Figure 1D. The data indicates that sixty per cent of questioned had higher education degree and only two respondents were characterized by primary education. Among the studied population, eight respondents were studying, whereas thirty-nine per cent completed secondary education level. Figure 1E presents the occupational profiles of surveyed individuals. Almost sixty per cent of respondents were white-collar workers, followed by both pensioners and physical laborers represented by 18 individuals. Among twelve students who participated in the survey, half of them apart from studying, were at the same time employed.

### 3.2. Dietary Habits during the COVID-19 Pandemic

#### 3.2.1. The Effect of the COVID-19 Pandemic on Changing Dietary Habits 

**Figure 2:**
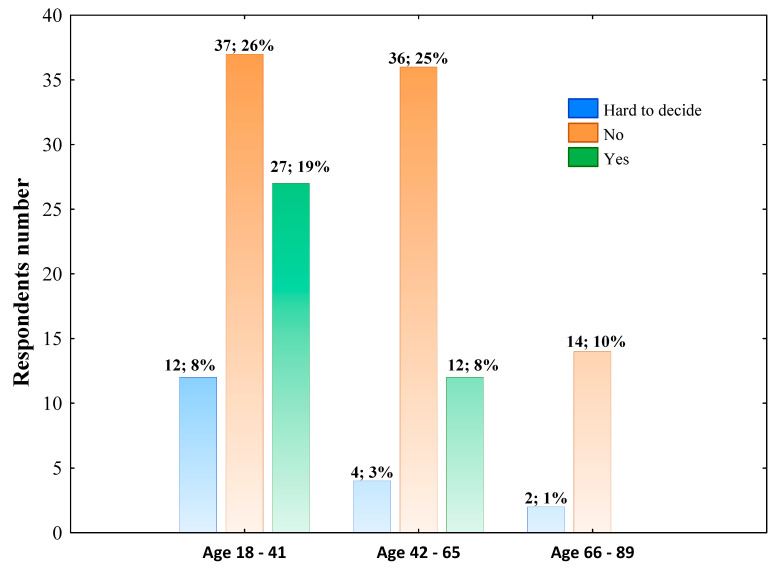
The effect of pandemic COVID-19 on changing dietary habits across age groups. The column description includes the respondents’ number aged in this range who chose such an answer, followed by the percentage of the total respondents. Data were analyzed using Pearson’s chi-squared test. Pearson’s χ^2^ = 12.604; df = 4; *p* = 0.0134; *n* = 144.

In general, 60% of respondents declared that the COVID-19 pandemic did not affect their dietary habits, whereas 26% of surveyed individuals answered in the affirmative. The effect of the COVID-19 pandemic on changing dietary habits was differentiated by age (Figure 2). This effect was not distinguished by gender (Pearson’s χ^2^ = 1.937; *p* = 0.37969), place of living (Pearson’s χ^2^ = 11.693; *p* = 0.16541), education level (Pearson’s χ^2^ = 13.598; *p* = 0.09286) and occupation (Pearson’s χ^2^ = 15.542; *p* = 0.21311).

Moreover, the multiple linear regression was conducted in the studied group to provide higher statistical power and demonstrate the independent influence of age on changing dietary habits during the COVID-19 pandemic. As is shown in Table 1, multivariate linear regression analysis revealed that, independent of the other factors considered (gender, education level and living place), individuals aged 66–89 declared changing dietary habits least often.

#### 3.2.2. The Effect of Lifestyle on the Number of Meals Consumed Daily during the COVID-19 Pandemic

The majority of the studied population, 61.38%, reported that they did not observe such an effect; 28.97% answered that eating more meals per day than before the COVID-19 pandemic and 4.14% responded that eating fewer meals per day than before pandemic. This tendency was not differentiated across age groups (Pearson’s χ^2^ = 9.578; *p* = 0.14356) nor by gender (Pearson’s χ^2^ = 6.581; *p* = 0.08651), and occupation (Pearson’s χ^2^ = 23.132; *p* = 0.18558). However, no such effect was more frequently pointed out by individuals with secondary (76.30%) and higher education levels completed (54.00%), while 34.50% of high educated respondents declared eating more meals than before pandemic (Pearson’s χ^2^ = 25.384; df = 12; *p* = 0.01311). Additionally, the multiple linear regression was conducted to evaluate the effect of lifestyle on the number of meals consumed daily, which revealed that, independent of the other factors considered, students, individuals with vocational, secondary, and higher education levels declared eating fewer meals per day least often (Table 2).

**Figure 3:**
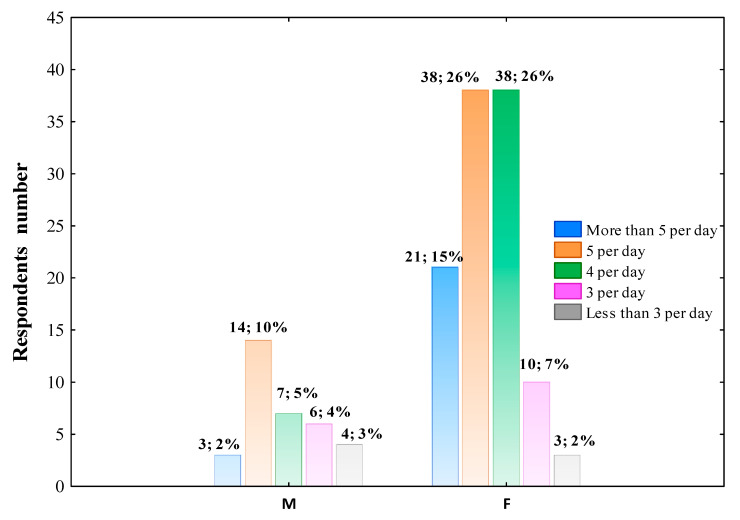
Meals consumption during the COVID-19 pandemic per day across gender groups (M–men; F–female). The column description includes the respondents’ number in this gender who chose such an answer, followed by the percentage of the total respondents. Data were analyzed using Pearson’s chi-squared test. Pearson’s χ^2^ = 9.653; df = 4; *p* = 0.0466; *n* = 145.

The analysis of questionnaires showed that the meals number consumed by respondents per day was differed across gender groups, as presented in Figure 3. Furthermore, the number of meals eating per day was differentiated by occupation (Pearson’s χ^2^ = 36.958; df = 24; *p* = 0.04418) but not by age (Pearson’s χ^2^ = 15.457; *p* = 0.05085, place of living (Pearson’s χ^2^ = 8.163; *p* = 0.94388), and education level (Pearson’s χ^2^ = 26.018; *p* = 0.05377).

#### 3.2.3. The Effect the COVID-19 Pandemic on Body Mass Change

**Figure 4:**
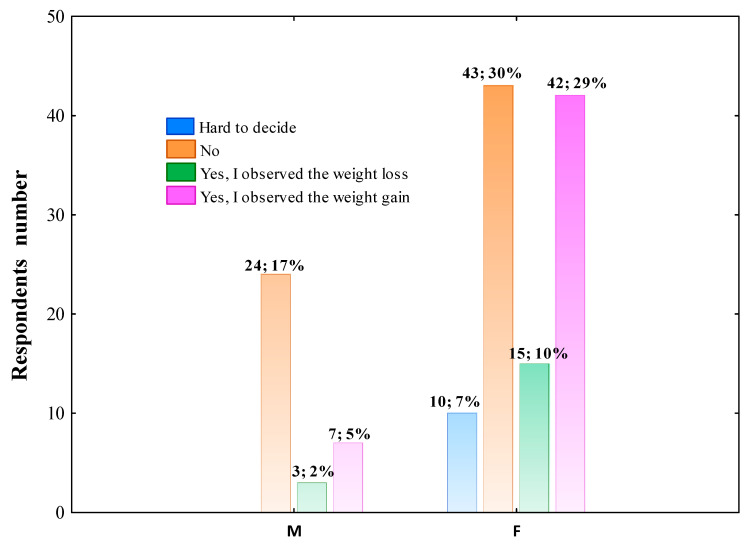
The effect of pandemic COVID-19 on body weight change across gender groups (M–men; F–female). The column description includes the respondents’ number in this gender who chose such an answer, followed by the percentage of the total respondents. Data were analyzed using Pearson’s chi-squared test. Pearson’s χ^2^ = 11.473; df =3; *p* = 0.0094; *n* = 145.

In the present study, 67 of the questioned population (46.21%) declared no weight change, whereas 49% of respondents (33.79%) marked an increase in body mass and 18 of participants (12.41%) reported a decrease in body weight during the COVID-19 pandemic. The data presented in Figure 4 shows the significant association between gender and the answer to the question relates to body mass change. Furthermore, the living place (Pearson’s χ^2^ = 21.905; df = 12; *p* = 0.03859), education level (Pearson’s χ^2^ = 22.818; df = 12; *p* = 0.02931) also differentiated this parameter. 

In the next step, multivariate linear regression analysis was performed in the whole group of surveyed individuals excluding those who marked ‘hard to decide’ to determine gender as the independent factor potentially influencing the body mass change. The data collected in Table 3 show that individuals with vocational and secondary education levels reported a decrease in body mass least often, independently of other factors considered, whereas hybrid working women declared most often an increase in body mass independent of age, education level and living place.

#### 3.2.4. The Effect the COVID-19 Pandemic on Sweets, Fast Foods, Sweet Drinks, Energy Drinks, Fruits, Vegetables and Fish Consumption 

**Figure 5:**
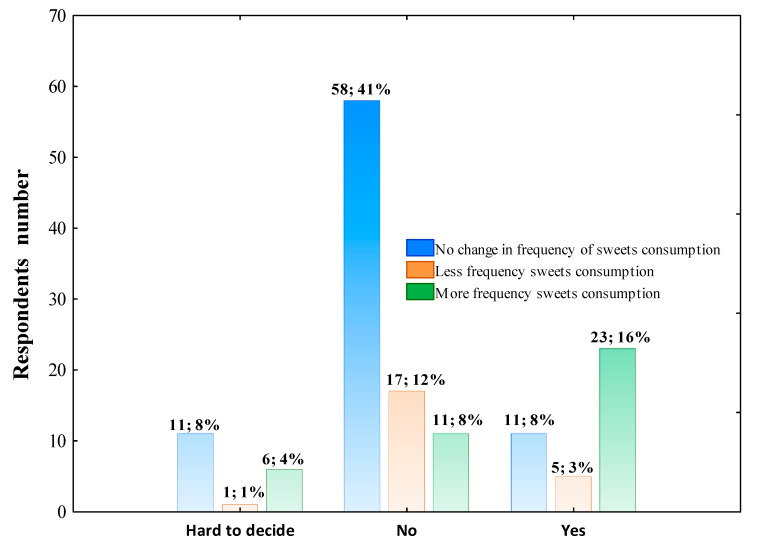
Sweets consumption frequency during the COVID-19 pandemic across respondents declared effect or no effect of pandemic on changing the dietary habits. The column description includes the respondents’ number who declared the effect or no effect of the pandemic on changing the dietary habits, followed by the percentage of the total respondents. Data were analyzed using Pearson’s chi-squared test. Pearson’s χ^2^ = 30.308; df = 4; *p* < 0.0000; *n* = 144.

The respondents were also asked whether the following food products—sweets, fast foods, sweet drinks, energetic drinks, fruits, vegetables and fish—were eaten by them more frequently, less frequently or at the same level per week during the COVID-19 pandemic than before. The statistical analysis did not show the association between gender, living place, occupation, and consumption frequency of all foods mentioned above per week. However, 32.18% of individuals with high education level marked eating sweets more frequently per week during the COVID-19 pandemic than before. In contrast, 58.62% of highly educated and 51.35% of secondary educated participants have not seen any changes (Pearson’s χ^2^ = 25.216; df = 8; *p* = 0.00143). The questionnaire’s analysis pointed out that 13 highly educated respondents declared more frequent fast foods consumption per week, 28 reported lower frequency, and 44 announced no changes, while up to 20 of respondents with secondary education level (62.50%) declared less frequency fast food consumption (Pearson’s χ^2^ = 15.736; df = 8; *p* = 0.04632). Furthermore, 25 highly educated respondents and 11 secondary educated respondents declared drinking sweetened beverages less frequently, whereas 55 of the first-mentioned group and 21 individuals with secondary education level declared no changes in sweet beverages drinking frequency (Pearson’s χ^2^ = 19.163; df = 8; *p* = 0.01401). The majority of individuals aged 18–41, 33, reported no effect of the COVID-19 pandemic on fast foods frequency consumption, whereas 25 and 17 declared less and more frequent consumption, respectively. The individuals aged 42–65 more often reported lower frequency of fast foods consumption (20 respondents) and 27 representatives did not note any changes (Pearson’s χ^2^ = 12.682; df = 4; *p* = 0.01294). The data presented in Figure 5 shows the frequency of sweets consumption among respondents who expressed their opinion on the effect of the pandemic on changing dietary habits. 

Additionally, logistic regression analysis was performed to identify whether and to what extent to which the surveyed respondents consumed sweets were more or less likely associated with the body mass change. As shown in Table 4, logistic regression analysis revealed that respondents who declared more frequency sweets consumption during the COVID-19 pandemic were more likely associated with an increase in body mass (OR = 6.75, 95% CI, 6.75–91.25), whereas individuals who reported fever frequency sweets consumption were more likely associated with weight loss (OR = 36.87, 95% CI, 7.53–247.10). 

#### 3.2.5. Supplementation of Vitamin D, C, and Mg. Pickled Food Consumption during the COVID-19 Pandemic

Supplementation of vitamin D, C, Mg and pickled products consumption were not differentiated by gender, age, living place, education level and occupation (Pearson’s χ^2^, *p* > 0.05 in all cases). However, 31 of 110 women respondents declared starting vitamin D supplementation, whereas 11 women, representing 10% of surveyed women, increased the dosage during the COVID-19 pandemic. The highest percentage of women, 34.55%, declared no change in dosage during the COVID-19 pandemic. Most of the men, 17 of 35, declared no supplementation of vitamin D. Twenty-seven of 18–41 aged respondents, representing 35.53% of this group and 18.6% of all the respondents, declared starting to take the vitamin D regularly during the COVID-19 pandemic. The declaration of vitamin D supplementation was not differentiated significantly across aged groups. However, the tendency to decrease in starting supplementation was found beginning from group aged 18–41, followed by 42–65 aged respondents, ending with group of age 66–85. Only fifteen women and three men declared an increase in vitamin C supplementation, whereas most of the surveyed answered that they do not supplement it at all. A similar tendency was found for Mg supplements-only 14 women and two men increased Mg supplements dosage during the COVID-19 pandemic, and 58% of surveyed women and 60% of surveyed men did not consume it at all. The majority of people surveyed, 84 women and 24 men, declared eating pickled products at the same level as before the pandemic. 

#### 3.2.6. Respondents’ Physical Activity and Daily Lifestyle during the COVID-19 Pandemic

**Figure 6:**
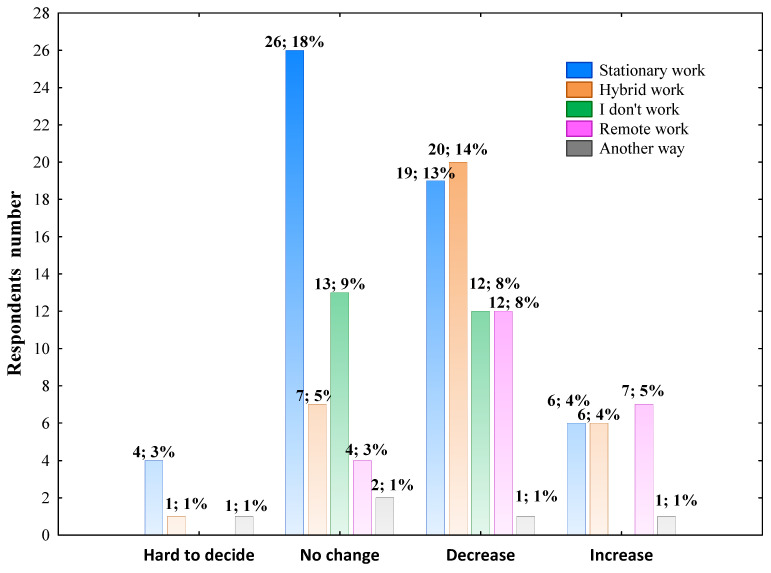
The effect of the COVID-19 pandemic on physical activity change according to the professional work mode. The column description includes the respondents’ number who declared the effect or no effect of the pandemic on changing physical activity, followed by the percentage of the total respondents. Data were analyzed using Pearson’s chi-squared test. Pearson’s χ^2^ = 26.850; df = 12; *p* = 0.00812; *n* = 144.

In general, 13.46% of surveyed reported an increase in physical activity, whereas 41.67% of the studied population declared decreased physical activity, and 33.33% of respondents answered that their physical activity has been kept at the same level as before the COVID-19 pandemic. The effect of the pandemic on physical activity was not differentiated by gender, age, living place and education level (Pearson’s χ^2^, *p* > 0.05 in all cases). The data presented in Figure 6 shows the significant association between professional work mode and the answer to the question relates to physical activity change (Pearson’s χ^2^ = 26.850; df = 12; *p* = 0.00812).

In the next step, multivariate linear regression analysis was performed in the whole group of surveyed individuals excluding those who marked ‘hard to decide’ to determine professional work mode as the independent factor potentially influencing the physical activity change. Most of the respondents working stationary declared no pandemic effect on the physi-cal activity change (Figure 6). In the present study, 20 hybrid working individuals, 19 stationary working respondents, and 12 remote working individuals declared a decrease in physical activity during the pandemic, which in the case of remote and hy-brid work, was independent of other factors such as age and gender (Table 5). The per-centage of surveyed individuals who reported that their level of physical activity in-creased during the COVID-19 pandemic was low across all the professional work mode groups. However, as shown in Table 5, the highest percentage of respondents who marked ‘My Level of Physical Activity Increased’ was people who declared remote work during the pandemic.

**Figure 7:**
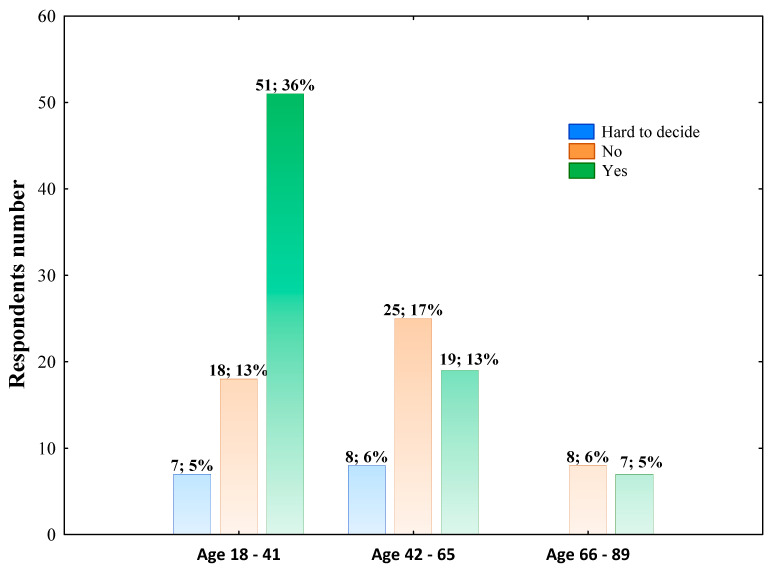
The change in daily lifestyle due to the COVID-19 pandemic across aged groups. The column description includes the respondents’ number aged in this range who chose such an answer, followed by the percentage of the total respondents. Data were analyzed using Pearson’s chi-squared test. Pearson’s χ^2^ = 15.015; df = 4; *p* = 0.00467; *n* = 144.

In general, 53.10% of respondents declared a change in daily lifestyle due to the COVID-19 pandemic, whereas 35.17% of surveyed did not observe such effect. However, 51 respondents aged 18–41 and 19 individuals aged 42–65 declared daily lifestyle changes and 7 of the 15 individuals belonging to 66–89 age group observed such effect (Figure 7). 

**Figure 8:**
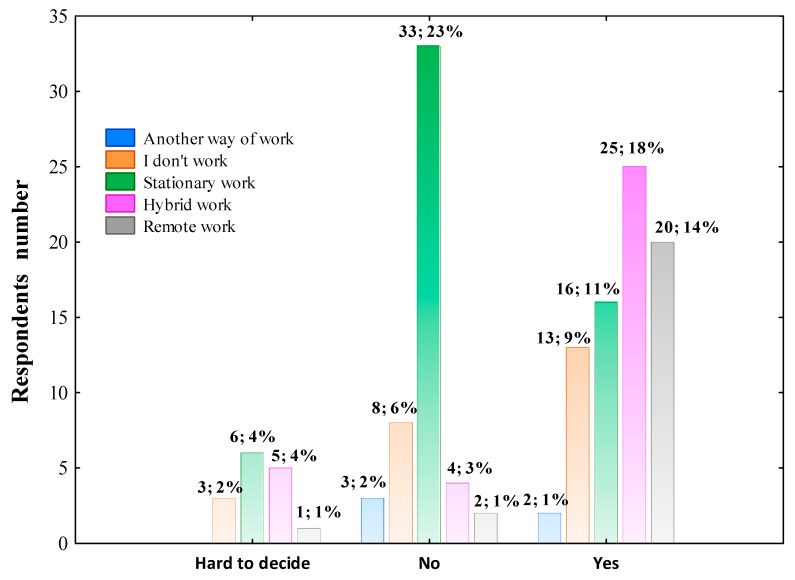
The effect of the COVID-19 pandemic on daily lifestyle change according to the professional work mode. The column description includes the respondents’ number who declared the effect or no effect of the pandemic on changing the daily lifestyle, followed by the percentage of the total respondents. Data were analyzed using Pearson’s chi-squared test. Pearson’s χ^2^ = 35.800; df = 8; *p* = 0.00002; *n* = 145.

Figure 8 presents that most of the individuals who declared hybrid working and remote working during the COVID-19 pandemic also observed the changes in daily lifestyle, whereas 23.00% of stationary workers did not notice such effect. 

In the next step, multivariate linear regression analysis was performed in the whole group of surveyed individuals excluding those who marked ‘hard to decide’ to determine professional work mode as the independent factor potentially influencing the daily lifestyle change. The data collected in Table 6 show that individuals who declared hydride work and remote work reported most often the effect of the COVID-19 pandemic on daily lifestyle change independent of age, gender, education level and living place.

## 4. Discussion

The present cross-sectional study showed that the COVID-19 pandemic impacted surveyed individuals’ dietary habits. In general, most respondents declared no effect of the COVID-19 pandemic on changing nutritional habits, whereby questioned aged 66–89 observed changing dietary habits least often. Eating more meals than before the pandemic declared about 34% of individuals with higher education levels completed. In contrast, individuals with vocational, secondary, and higher education levels declared eating fewer meals per day least often. This may result from professional workstyle, decreased or increased stress, daily lifestyle change during the COVID-19 pandemic.

The present study results reveal expected changes in the daily lifestyle of questioned participants, especially those aged 18–41. The results are not surprising because of schools and universities closures, the lack of face-to-face interaction with peers and co-workers, increased stress for many families and forced change of professional work mode [17]. Moreover, the effect of the COVID-19 pandemic on daily lifestyle reported by the majority of individuals who worked hybrid and remote system is an evidence that such modification of workstyle turned out to be one of the most significant change in many people’s lives. The main finding of this study was that individuals who declared hydride work and remote work reported most often the effect of the COVID-19 pandemic on daily lifestyle change independent of age, gender, education level and living place. It should be emphasized that such work modes were not standard in Poland before the COVID-19 and we wonder if both work modes catch on eventually. 

We also found out that the impact of the pandemic on daily lifestyle was also associated with body mass increase. In fact, women issued such declarations more frequently than men. Moreover, we found that individuals with vocational and secondary education levels reported a decrease in body mass least often, whereas hybrid working women declared most often an increase in body mass independent of age, education level and living place. Błaszczyk-Bębenek et al. demonstrated that the body weight of 32% of the surveyed did not change during isolation, whereas the weight of almost 46% of the respondents significantly increased [18]. The tendency percentage found in the present study is different from those mentioned above, and probably results from the lower number of our study’s participants. Some studies pointed out that women are less resistant to stress and a subconscious reaction to reduce stress and anxiety is snacking and more frequent eating [14,19]. In the present study, although almost 43% of questioned women declared the body mass increase, nearly 43% of surveyed women reported no change in body weight, whereas 15% reported body mass loss. In our opinion, the results are positive and may suggest an increased time for meals preparing, more time for celebrating eating with family, eating meals at the fixed time and desire to change dietary habits. In addition, it is worth mentioning that increased weight, especially obesity, is one of the most critical risk factors of more severe COVID-19 disease course and fatality [18,20]. Despite the low number of male participants, the results show that most of this study group declared no change in the body mass during the COVID-19 pandemic. It is interesting, considering that most studies reported poorer dietary habits and a higher frequency of overweight and obesity among men compared to women [18]. 

The positive results of social isolation manifested in eating behaviors cannot also be overlooked. Some studies stated the decreases in alcohol binge drinking, decreases in eating fried foods and an increase in eating fresh foods [18,21,22]. In the present study, we have not found the significant impact of the COVID-19 pandemic on increase in eating fruits, vegetables and fish per week. In general, almost 56% of all respondents declared no change in frequency of sweets consumption, whereas 28% reported eating sweets more frequently per week during the COVID-19 pandemic than before. Our results show that almost one-third of highly educated individuals declared more frequent sweets consumption per week than before the pandemic. It may be associated with evaluated stress and easier availability to sweet products because of hybrid or remote work, which were declared by individuals belonging to this study subgroup. On the other hand, the surveyed who stated no influence of the pandemic on the bodyweight more often revealed no changes in sweets, fast foods, sweet drinks, fruits and vegetable consumption frequency. Sidor and Rzymski found that nearly one-third of 1097 respondents consumed sweets at least every day and the same proportion did not consume fresh vegetables daily [22]. In our study, eating more sweets and fruits was most frequently reported by respondents who observed the effect of the COVID-19 pandemic on their dietary habits and stated weight gain during the COVID-19 pandemic. We also found that respondents who declared more frequency sweets consumption during the COVID-19 pandemic were more likely associated with an increase in body mass. The beneficial health effects of fruit, including attenuation of obesity, diabetes and coronary heart disease are well documented [23,24]. Despite the identified anti-obesity effect of fruit, some studies have also reported the pro-obesity impacts of certain types of fruit [24,25]. In the light of recent studies, it should be mentioned eating fruit can increase calorific intake and positively impact energy homeostasis to promote obesity eventually, which most consumers are not aware of.

In our opinion, physical activity is one of the essential components of daily lifestyle that showed the most substantial negative influence of the COVID-19 pandemic. The present study results show that individuals who declared remote work and hybrid work reported a decrease in physical activity most often, whereas an increase in physical activity was stated most often by individuals who performed remote work.

The scientific world had high hopes for the possible vitamin D protective effect against the COVID-19 disease. The present study has not confirmed the hypothesis that the COVID-19 pandemic increased vitamin D consumption in surveyed individuals. Interestingly, Puścion-Jakubik et al. demonstrated that a significantly greater percentage of Polish respondents declared not taking food supplements with zinc and vitamin D during the first wave of the COVID-19 pandemic [26]. In many countries, an evaluated interest in diet supplementation during the COVID-19 pandemic was found [27,28,29]. Our results demonstrate higher intake of vitamin D by women than by men. It may suggest that women are more worried about their health and more interested in pro-health prophylaxis than men. The Polish Economic Institute notified that the worth of the food supplements market was estimated to amount to 4.4 billion PLN in 2017 and over 70% of Poles used food supplements [26,30]. It seems that cognizance in differences between medications and supplements, side effects, and their possible interaction with medications has increased. It is not excluded that most of the respondents did not decide to start vitamin D supplementation or increase the dosage by themselves, without recommendation. It is worth emphasizing, that consulting doctors or pharmacists was strongly limited, especially during the first and second waves of the COVID-19 pandemic in Poland. Both aspects were analyzed by Puścion-Jakubik et al. [26]. They found that most respondents reported that food supplements could have side effects and be overdosed. In our study, the effect of age on the increase in vitamin D supplementation has not been found. Our results point out the importance of another aspect of this issue, namely vitamin D supplementation, which before the COVID-19 pandemic, was used already by 50% of all women. Our results may indicate the increasing knowledge on the evidence of vitamin D’s health benefits as well as vitamin D-related diseases, such as depression and osteoporosis, commonly encountered among older women.

Additionally, we have not found the effect of gender, age, living place, education level and occupation on supplementation of vitamin C, and Mg during the COVID-19 pandemic. It suggests that negative emotions related to the COVID-19 pandemic did not result in starting Mg supplementation or increasing dosage in the studied group. In the present study, it has been observed that gender, age, living place, education level and occupation had not contributed to the frequency of consumption of pickled products. It seems that the tradition of pickling and fermenting food is so widespread and deeply rooted in Poland that the COVID-19 pandemic did not increase such tendency significantly.

## 5. Limitations

In the present study, some limitations need to be underlined. The first limitation is related to the inequitable gender proportions of the study group. Next is associated with the low number of participants aged higher than 60. Unfortunately, the inadequate response of men is a pretty common problem of questioned-based surveys. Moreover, the COVID-19 pandemic forced performing the study online, which has likely limited seniors’ participation. Due to the restricted social contacts, we could not make anthropomorphic measurements by ourselves, including height and weight, and we had to base on participants-reported data. We assume that if the size of the study group was increased and a similar number of men to women and more seniors answered the questions, the results could be different. 

## 6. Conclusions

The results of the present study showed the COVID-19 pandemic had an influence on surveyed individuals’ eating habits, physical activity and lifestyle. The dietary habits change was declared least often by individuals aged 66–89. A majority of surveyed women declared the body mass change, and the living place and educational level also differentiated this parameter. A significant percentage of individuals who experienced modification of dietary habits, also reported eating fruits and sweets more frequently. Their weight changed as well. No increase in the consumption of vitamin D, C and Mg supplements and pickled products was found. The effect of COVID-19 on lifestyle change was more frequently reported and was associated with the respondents’ age and professional work mode. 

## Figures and Tables

**Table 1:** Multivariate linear regression analysis of the association between age, gender, education level, living place and changing dietary habits in studied group.

Parameter	Coefficient	*p*
age 42–65	−0.0818	0.3673
age 66–89	−0.3124	0.0394 *
gender (female)	0.0247	0.8065
education level (secondary)	0.2525	0.5854
education level (vocational)	0.3701	0.4536
education level (higher)	0.4768	0.2957
education level (student)	0.3966	0.4155
living place (<50,000)	−0.1524	0.3474
living place (50,000–100,000)	−0.3363	0.1219
living place (100,001–250,000)	0.2689	0.2633
living place (>250,000)	−0.0883	0.5734

The reference level of analyzed categorical variables are: age 18–41, gender (male), education level (primary), living place (country), respectively. * statistically significant.

**Table 2:** Multivariate linear regression analysis of the association between age, gender, education level, living place and the effect of lifestyle on the number of meals consumed daily during the COVID-19 pandemic.

	Yes, I Eat Fewer Meals per Day Than before the COVID-19 Pandemic		Yes, I Eat More Meals per Day Than before the COVID-19 Pandemic	
Parameter	Coefficient	*p*	Coefficient	*p*
age 42–65	−0.0662	0.3312	−0.0058	0.9489
age 66–89	−0.1521	0.1160	−0.0436	0.7678
gender (female)	−0.1151	0.1227	0.1197	0.2332
education level (secondary)	−0.9933	0.0010 *	−0.7364	0.1193
education level (vocational)	−1.029	0.0014 *	−0.8378	0.0974
education level (higher)	−0.8906	0.0025 *	−0.5741	0.2172
education level (student)	−1.1080	0.0009 *	−0.4657	0.3415
living place (<50,000)	0.1629	0.2882	−0.2243	0.1973
living place (50,000–100,000)	0.2092	0.2912	−0.0631	0.7761
living place (100,001–250,000)	0.0503	0.8470	0.2615	0.2971
living place (>250,000)	0.0570	0.7072	−0.108	0.5137

The reference level of analyzed categorical variables are: age 18–41, gender (male), education level (primary), living place (country), respectively. * statistically significant.

**Table 3:** Multivariate linear regression analysis of the association between age, gender, education level, living place and the effect of pandemic COVID-19 on body weight change.

	Yes, I Have Noticed Weight Loss		Yes, I Have Noticed an Increase in Body Weight	
Parameter	Coefficient	*p*	Coefficient	*p*
age 42–65	−0.0313	0.5977	−0.0487	0.6373
age 66–89	0.0848	0.3866	0.2191	0.2678
gender (female)	−0.0252	0.7049	0.2417	0.0385 *
education level (secondary)	−1.034	<0.0001 *	−0.0563	0.8752
education level (vocational)	−1.099	0.0001 *	−0.2701	0.5230
education level (higher)	−0.9205	0.0003 *	−0.0029	0.9931
education level (student)	−0.9634	0.0008 *	−0.0434	0.9140
living place (<50,000)	0.1230	0.3439	−0.0792	0.6648
living place (50,000–100,000)	−0.0269	0.8773	0.0463	0.8488
living place (100,001–250,000)	0.0490	0.8266	0.3734	0.3210
living place (>250,000)	0.0153	0.9056	−0.1274	0.4661
professional work mode (I don’t work)	−0.0517	0.4933	−0.1564	0.3329
professional work mode (remote work)	0.0342	0.7226	0.2197	0.1166
professional work mode (hybrid work)	−0.0482	0.5429	0.2775	0.0311 *

The reference level of analyzed categorical variables are: age 18–41, gender (male), education level (primary), living place (country), professional work mode (stationary work), respectively. * statistically significant.

**Table 4:** Odds ratio for type of change in body mass (weight gain, weight loss or unchanged) during the COVID-19 pandemic by sweet consumption factor.

		Weight Loss				Weight Gain			
Factor		ORs	95% CI			ORs	95% CI		
			Lower Limit	Upper Limit	*p*		Lower Limit	Upper Limit	*p*
Sweets consumption	More sweets	4.53	0.49	34.04	0.1443	22.21	6.75	91.25	<0.0001 *
	Less sweets	36.87	7.53	274.10	<0.0001 *	3.05	0.66	14.28	0.1468
	No change (reference)	1	-	-	-	1	-	-	-

ORs are adjusted for age, sex and professional work mode. * statistically significant.

**Table 5:** Multivariate linear regression analysis of the association between age, gender, professional work mode, and the effect of pandemic COVID-19 on the physical activity change.

	My Level of Physical Activity Decreased		My Level of Physical Activity Increased	
Parameter	Coefficient	*p*	Coefficient	*p*
age 42–65	0.0243	0.8120	−0.0913	0.4246
age 66–89	−0.1883	0.2860	−0.0539	0.7458
gender (female)	−0.0343	0.7555	0.2196	0.0788
professional work mode (I don’t work)	0.1397	0.3199	−0.2090	0.1818
professional work mode (remote work)	0.3298	0.0286 *	0.4161	0.0068 *
professional work mode (hybrid work)	0.3263	0.0074 *	0.2656	0.0661

The reference level of analyzed categorical variables are: age—18–41, gender—(male), and professional work mode (stationary work), respectively. * statistically significant.

**Table 6:** Multivariate linear regression analysis of the association between age, gender, education level, living place, professional work mode and daily lifestyle change.

Parameter	Coefficient	*p*
age 42–65	−0.1624	0.0651
age 66–89	−0.2825	0.0735
gender (female)	−0.0164	0.8691
education level (secondary)	0.1334	0.6726
education level (vocational)	0.2887	0.4365
education level (higher)	0.2193	0.4745
education level (student)	0.2343	0.5008
living place (<50,000)	−0.2326	0.1634
living place (50,000–100,000)	0.0159	0.9443
living place (100,001–250,000)	−0.0131	0.9557
living place (>250,000)	−0.1245	0.4383
professional work mode (I don’t work)	0.3284	0.0178 *
professional work mode (remote work)	0.4815	0.0001 *
professional work mode (hybrid work)	0.4747	<0.0001 *

The reference level of analyzed categorical variables are: age 18–41, gender (male), education level (primary), living place (country), and professional work mode (stationary work), respectively. * statistically significant.

## Data Availability

The datasets used and/or analyzed during the present study are available from the corresponding author on reasonable request.
